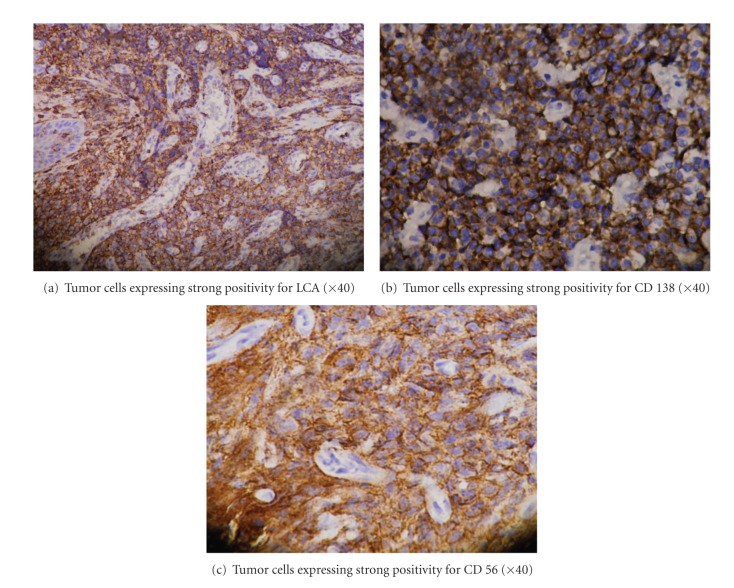# Plasmablastic Lymphoma of Gingiva Mimicking a Reactive Lesion: A Case Report

**DOI:** 10.1155/2012/259307

**Published:** 2012-09-13

**Authors:** Neeta Bagul, G. S. Mamatha, Aditi Mahalle

**Affiliations:** Department of Oral Pathology and Microbiology, Dr. D. Y. Patil Dental College and Hospital, Pimpri, Pune 411018, India

## Abstract

Oral plasmablastic lymphoma (PBL) is a rare malignancy, associated with HIV or other immunocompromised conditions. The lesion constituted a new subtype of diffuse large B-cell lymphoma and proposed a distinct entity based on its basic morphology, its clinical behaviour involving predominantly extramedullary sites (particularly oral cavity), and its limited antigenic phenotype data suggesting plasmacytic differentiation. Authors here report a case of apparently healthy individual aged 35 years, presenting one-month history of swelling associated with loosened teeth around upper anteriors. Following incisional biopsy, routine histopathologic and immunohistochemical studies, the diagnosis of plasmablastic lymphoma was given.

## 1. Introduction 

Plasmablastic lymphoma is a unique AIDS-related lymphoma, which was first described in the jaws and oral cavity of HIV-infected persons [1, 2]. The disease accounts for 2.6% of all HIV-related non-Hodgkin lymphomas [3]. The lymphoma has also been reported in HIV-negative persons, particularly those who have immunosuppression. The lymphoma is listed in the World Health Organization 2001 classification as a variant of diffuse large B-cell lymphoma [4].

It usually develops in middle-aged adults [4], but can also occur in the pediatric age group [5]. The lymphoma involves predominantly the gingival and palatal mucosa, causing thickening and ulceration with a tendency to infiltrate adjacent bone [3]. The clinical appearance may mimic periodontal disease, Kaposi sarcoma, or melanoma [6]. Radiographic changes include widening of the periodontal ligament space and loss of the lamina dura [7].

Plasmablastic lymphoma of oral mucosa contains a monomorphic population of plasmablasts with no or minimal plasmacytic differentiation. The histological findings of a diffuse infiltrative growth pattern, brisk mitotic activity, and necrosis, along with the fact that they are rapidly growing destructive tumors, supports their designation as a high-grade malignant lymphoma.

Plasmablasts are lymphoid cells that morphologically resemble B-cell immunoblasts but have acquired a plasma cell immunophenotype (i.e., loss of B-cell markers and surface immunoglobulin with the acquisition of plasma cell surface markers). Thus, unlike immunoblasts, plasmablasts fail to express CD45 (leukocyte common antigen) as well as the B-cell marker CD20 and are only variably immunoreactive for CD79a—a broader-spectrum B-cell marker. They are also negative for pan-T-cell markers. Positive staining for plasma cell markers such as VS38c, CD38, MUM-1, and CD138 indicates a phenotype akin to plasma cells [8, 9]. Newer B-lineage markers (e.g., OCT.2 and BOB.1) may prove useful in determining a B-cell origin in plasmablastic lymphomas [10, 11].

Treatment includes radiotherapy, chemotherapy, surgery, or a combination of these modalities. The lymphoma is known to be rapidly progressive with a poor prognosis for persons with HIV/AIDS, with a median survival of 6 months. After the institution of highly active antiretroviral therapy, an increase in survival time has been noticed [12–14].

## 2. Case Report

A 35-year-old, apparently healthy individual reported to the Department of Oral Pathology complaining of a painless growth in the upper jaw since 1 month that gradually grew in size. No other symptoms were reported before the onset of the swelling. The patient did not notice loosening of the teeth associated with the growth. Extraorally no abnormalities were detected There was no history of trauma or spontaneous bleeding. The patient did not give a history of tobacco smoking, alcohol consumption, or drug use.

**Figure 1:**
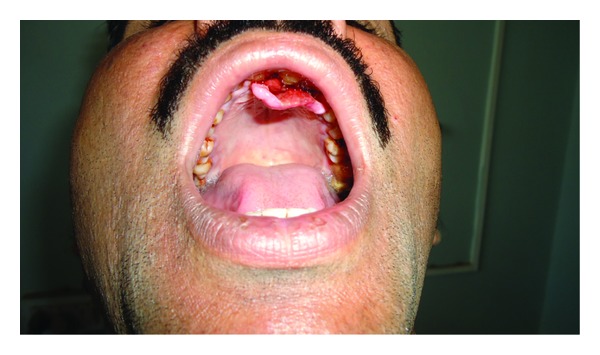
Exophytic gingival growth in the maxillary anterior palatal region.

Intraoral hard tissue examination revealed an exophytic, lobulated mass, irregular in consistency, in the maxillary palatal aspect extending from the right maxillary lateral incisor to the left maxillary first premolar region. Ulcerated growth was noted with the palatal aspect of 11, 12, 21, and 22. The growth was soft in consistency, and bleeding on probing was evident (Figure 1).

Routine haematological examinations were carried out which revealed normal numbers of red blood cells and white blood cells. Platelet count was also within normal limits. The HIV status of the patient was negative.

**Figure 2:**
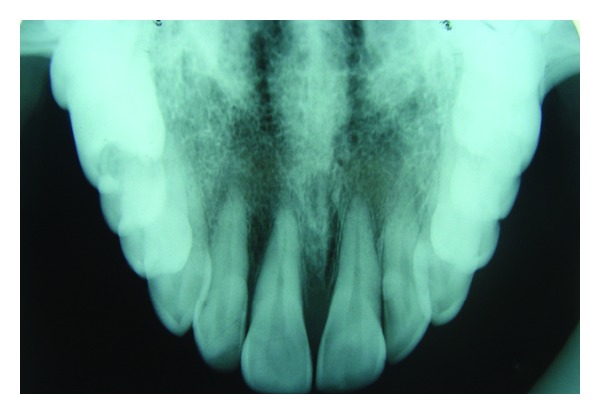
Interdental bone loss in relation to maxillary central and lateral incisors.

The radiographic investigations included occlusal and intraoral periapical radiography of the anterior part of the upper jaw which revealed interdental bone loss with 11, 21 and 21, 22 (Figure 2).

**Figure 3:**
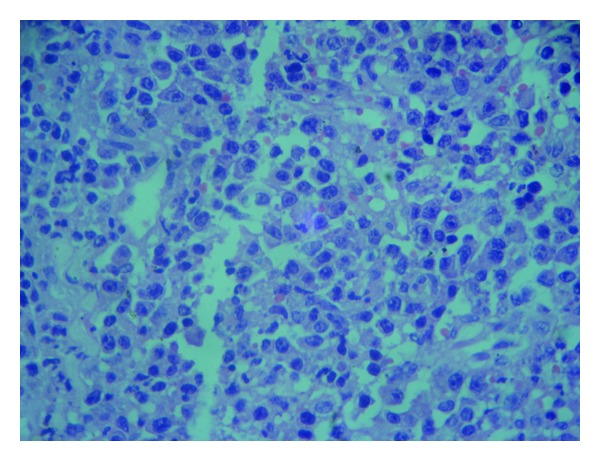
Diffuse large lymphoid cells with abundant eosinophilic cytoplasm, centrally or few eccentrically placed nuclei, and pleomorphic basophilic nucleoli (×40).

An incisional biopsy of intraoral mass was performed. Histopathological examination of haematoxylin and eosin stained section showed covering of parakeratinized stratified squamous epithelium with ulceration at places. There was no evidence of dysplasia in the epithelium. Underlying connective tissue stroma revealed diffusely arranged large round tumor cells. Majority of the tumor cells showed open face nuclei with prominent nucleoli, while few others showed eccentric nuclei (Figure 3). The distribution of nucleoli was located either in the center or peripherally near the nuclear membrane. Admixed among these tumor cells, macrophages were also seen. On immunohistochemical analysis, tumor cells were positive for LCA, CD-138, and CD-56 (Figures 4(a), 4(b), and 4(c)) and negative for CD20, CD30, ALK1, Cyclin D1, and EMA.

## 3. Discussion

Non-Hodgkin's lymphoma finds its mention in oral manifestations of HIV/AIDS for the first time in Pindborg's classification [15], and plasmablastic lymphoma was first described by Delecluse et al. in 1997 [1]. Originally it was described to be a disease specifically involving the oral cavity of immunodeficient patients, but, number of cases have been reported in various extraoral sites including nasopharynx, stomach, small bowel, anus, lungs, skin, and so forth. Some of the cases are even reported in immunocompetent patients [16].

Oral NHL may appear as swelling, ulceration, exophytic masses, delayed healing of extraction sites, or trigeminal neuropathy. Recognition of this distinctive type of lymphoma confined to gingiva is important to avoid confusion with other gingival enlargements as PBL may mimic benign/reactive gingival enlargements like pyogenic granuloma and peripheral giant cell granuloma [17].

Thus, it is imperative to include PBL in the differential diagnosis of solitary gingival enlargement, as seen in our case.

The morphological hallmark of plasmablastic lymphoma is diffuse submucosal proliferation of monomorphic large-sized tumor cells with deep ulceration of the overlying mucosa. The tumor cells typically demonstrate high nuclear-cytoplasmic ratio, moderate amount of amphophilic, or basophilic cytoplasm with squared or rounded borders and centrally or eccentrically placed round nucleus with smooth nuclear outlines [18]. Plasmablasts are a type of lymphoid cells that have retained the morphology of an immunoblast but have already acquired the immunophenotype of a plasma cell [1]. Plasmablastic lymphoma may show plasma cells, but these are always reactive in nature and never neoplastic. Morphologically, a neoplastic plasma cell population is characterized by an intimate admixture of mature plasma cells with varying proportion of bi/multinucleated pleomorphic and immature plasma cells at all stages of maturity forming a sort of morphological continuum [2].

Kane et al. proposed minimum morphological criteria to diagnose plasmablastic lymphoma which includes the following. Predominant population of plasmablasts which are large monomorphic cells with high nuclear: cytoplasm ratio, moderate amount of amphophilic cytoplasm, and round nucleus with prominent central nucleolus; high mitotic and/or apoptotic index; absence of neoplastic plasma cells in the background [18].

PBL patients have been treated heterogeneously, and well-defined guidelines are lacking. Chemotherapy, radiotherapy with or without surgical excision, has been reported with various degrees of success [19, 20].

The prognosis of PBL is reported to be poor with or without treatment, and death is predicted in 1–24 months with average survival time of 6 months, though it has been suggested that addition of highly active antiretroviral therapy (HAART), chemotherapy is capable of significantly improving the prognosis [20].

## 4. Summary

In conclusion, this paper has detailed the case of plasmablastic lymphoma clinically mimicking a reactive lesion of gingiva; PBL should be considered in differential diagnosis of gingival enlargements. Patients with gingival enlargements are seen very commonly in daily clinical practice, but to differentiate neoplastic lesions from nonneoplastic/reactive lesions is essential in treating planning. Biopsy of these gingival enlargements, histopathological examination, and immunohistochemical analysis will aid in accurate diagnosis and treatment. Patient was referred to the higher centre after the final diagnosis.

## Figures and Tables

**Figure 4:**